# Contribution of TGF-Beta-Mediated NLRP3-HMGB1 Activation to Tubulointerstitial Fibrosis in Rat With Angiotensin II-Induced Chronic Kidney Disease

**DOI:** 10.3389/fcell.2020.00001

**Published:** 2020-02-05

**Authors:** Kaimin Zhang, Chun Fan, Dongpeng Cai, Yi Zhang, Rui Zuo, Li Zhu, Yue Cao, Jian Zhang, Chao Liu, Yang Chen, Hui Liang

**Affiliations:** ^1^School of Pharmaceutical, Guangzhou University of Chinese Medicine, Guangzhou, China; ^2^Guangdong Provincial Hospital of Chinese Medicine, Guangzhou University of Chinese Medicine, Guangzhou, China; ^3^Hubei Key Laboratory of Diabetes and Angiopathy, Hubei University of Science and Technology, Xianning, China; ^4^Department of Nephrology, Guangdong Provincial Hospital of Chinese Medicine, Guangzhou, China

**Keywords:** chronic kidney disease, Ang II, TGF-beta, fibrosis, NLRP3 inflammasome

## Abstract

Fibrosis is a common phenotype that often leads to the progression of blood pressure-induced chronic kidney disease (CKD). TGF-beta plays an important role in promoting pathogenesis, and NLRP3 is a critical mediator in the progression of blood pressure-induced CKD. However, the pathophysiological roles of the TGF-beta-mediated NLRP3 pathway in modulating fibrosis in blood pressure-induced CKD have not been elucidated. The present study aims to investigate the contribution of TGF-beta-mediated NLRP3 inflammasome to renal fibrosis in rats with high blood pressure. By treating rats with angiotensin II (Ang II) for 14 days, we observed the development of fibrosis, characterized by epithelial–mesenchymal transition (EMT) markers [alpha-smooth muscle actin (alpha-SMA), MMP-2, and MMP-9]. Immunohistochemical analysis further revealed that TGF-beta and NLRP3 inflammasome activation [high-mobility group box 1 (HMGB1), IL-1beta, and NLRP3] were significantly upregulated in the kidney of rats with Ang II-induced hypertension. Interestingly, we observed that Ang II could not increase the production of NLRP3 proteins, but TGF-beta could induce NLRP3 protein expression in cultured NRK-52E cells. Furthermore, we speculated that TGF-beta played a pathogenic role in Ang II-induced CKD because TGF-beta induced the activation of NLRP3 inflammasomes and Gasdermin D cleavage expression. We also proved that the pharmacological inhibition of NLRP3 by ISO caused a decrease in TGF-beta-induced NLRP3 inflammasome activation and the expression of EMT markers (alpha-SMA and CollagenI) and Gasdermin D cleavage. Collectively, these results suggest that TGF-beta-mediated NLRP3 inflammasome activation may cause the release of HMGB1 and an increase in Gasdermin D cleavage in NRK-52E, thereby contributing to renal fibrosis in Ang II-induced CKD. These findings provide novel insights into the pathogenic role of NLRP3 in CKD associated with high blood pressure.

## Introduction

Chronic kidney disease (CKD) is considered a worldwide health issue that affects about 5–10% of the adult population (Bowers et al., 2019). The high risk of this disease results in a high mortality rate. Although tremendous progress has been made in exploring the molecular mechanisms and signaling pathways of CKD in recent years, the event that initiates this process has not been elucidated. Studies have confirmed that hypertension, proteinuria, obesity, diabetes, and dyslipidemia (Richter et al., 2015; Amador-Martinez et al., 2019; Ku et al., 2019) can lead to progressive fibrosis, which generally refers to the excessive deposition of extracellular matrix components in the tubulointerstitium, resulting in the loss of renal function during CKD. Angiotensin II (Ang II) plays a critical role in hypertension-induced fibrogenic mechanisms. It is the main effector of the renin angiotensin aldosterone system (RAAS), and increasing its levels can promote RAAS activity, resulting in severe vascular, glomerular, and tubulointerstitial injuries along with the release of the cytokine TGF-beta through the angiotensin type 1 receptor (Balakumar et al., 2019). TGF-beta is required for Ang II to activate fibroblasts and induce fibrosis (Ehanire et al., 2015b; Angelov et al., 2017). In CKD, targeting fibrotic progression may reduce kidney injury and improve the efficiency of treatment strategies based on cellular studies. Therefore, slowing down fibrosis progression or accelerating its reversal can be a good approach for the treatment of CKD.

Fibrosis is a common outcome of CKD that is always accompanied by the development of inflammation. In general, the inflammatory response is stimulated by renal fibrotic lesions. However, aggravated inflammation further accelerates the progression of fibrosis. The role of inflammation in the kidney fibrogenic response has not been elucidated. The NLRP3 inflammasome plays a significant role in inflammation by regulating the maturation of proinflammatory cytokines, such as IL-1beta and IL-18. NLRP3 is best known as a functional component of the inflammasome. Pathogen-associated molecular patterns or damage-associated molecular patterns trigger NLRP3 to interact with the adapter protein apoptosis-associated speck-like protein (ASC) through pyrin domain (PYD)–PYD interactions, which form a nucleation platform of ASC filaments. The caspase recruitment domain (CARD) on pro caspase-1 binds to CARD on the surface of the ASC filament (Chang et al., 2014; Groslambert and Py, 2018; Ranson et al., 2018), finally forming the NLRP3 inflammasome and leading to the maturation of proinflammatory cytokines IL-1β and IL-18 (Lian et al., 2018). NLRP3 inflammasome and caspase-1 activation are the key mediators in the progression of fibrosis. Considerable evidence has revealed a strong association between renal function and NLRP3. The NLRP3 knockout can attenuate renal dysfunction in a unilateral ureteral obstruction (UUO) model of CKD, improve renal function, and alleviate inflammation and the level of CTGF (TGF-β1 and connective tissue growth factor) in STZ-induced diabetic mice (Guo et al., 2017; Wu et al., 2018). The use of a specific inhibitor of the NLRP3 inflammasome CP-456,773 can prevent kidney dysfunction in a murine model of crystal nephropathy (Ludwig-Portugall et al., 2016). Correa-Costa et al. (2011) reported the upregulation of NLRP3 mRNA in the biopsies of human kidneys from a large number of patients with different kinds of kidney diseases, such as IgA nephropathy, hypertensive nephrosclerosis, minimal change disease, focal segmental glomerulosclerosis, and acute tubular necrosis.

TGF-beta is the most potent profibrotic cytokine in Ang II-related CKD, which has been reported that the role of TGF-beta signaling in fibrosis is associated with NLRP3 inflammasome (Wang et al., 2014; Qiu and Tang, 2016). The Muruve lab proposes a regulatory role of NLRP3 on TGF-β signaling in tubular epithelial cells. With the stimulation of TGF-beta, the expression of the NLRP3 protein was significantly increased in a Smad3-dependent manner. TGF-β-mediated Smad2 and Smad3 phosphorylation are also related to NLRP3 and ASC. As an element of epithelial–mesenchymal transition (EMT) of tubular epithelial cells, NLRP3 is associated with tubular atrophy and progressive interstitial fibrosis in CKD (Lorenz et al., 2014). Moreover, TGF-beta induced EMT was associated with an increased protein level of NLRP3, apoptosis-associated speck-like protein containing a CARD (ASC), and pro caspase-1 in human peritoneal mesothelial cells (HPMCs), with an upregulated production of IL-1beta and IL-18, which was inhibited by the gene silencing of NLRP3/ASC with siRNA, caspase inhibitors, or the neutralization of IL-1beta/IL-18 (Ko et al., 2019). Currently, studies on the impact of TGF-beta-mediated inflammation on renal diseases are mainly focused on hypertension (Felix et al., 2019; Southgate et al., 2019), and reports on TGF-beta-mediated NLRP3 inflammasomes and renal fibrosis are limited.

In the present study, we investigated the relationship between the NLRP3 inflammasome and fibrosis in chronic kidney injuries. Our study further proves the role of TGF-beta-mediated NLRP3 inflammasomes in CKD-related fibrosis and provides evidence to help elucidate the underlying mechanisms of action.

## Materials and Methods

### Animal Models

Angiotensin II (Sigma, 200 ng/kg min) was infused into uninephrectomized rats for 14 days using Alzet osmotic minipumps (Model 2002, Cupertino, CA, United States) that were transplanted subcutaneously. The left kidney from the flank region was exposed, and the interstitial infusion catheter was placed into the renal medulla, which is approximately 4–5 mm underneath the surface of the kidney, and bonded using 3 mol/L Vetbond tissue adhesive (3M, Saint Paul, MN, United States) with a small piece of fat tissue (Wang et al., 2017). After the experiment, the kidneys were removed and cut longitudinally. Part of the kidney was fixed in 4% paraformaldehyde and stored at room temperature. All animal studies were approved by the Animal Ethics Committee of the Guangzhou University of Chinese Medicine.

### Cell Culture

NRK-52E cells were maintained in DMEM with 10% fetal bovine serum (FBS) at 37°C in 5% CO_2_ and passaged twice a week. Cells were seeded into six-well plates (1.5 × 10^5^ per well). After the cells adhered, the medium was removed and 1 ml of 10% FBS DMEM was added in the presence of Ang II (100 and 1000 nM) or TGF-beta (5 and 10 ng/ml) for 24, 48, and 72 h. NRK-52E cells were also treated with the NLRP3 inhibitor ISO (4 μg/ml) or AV-YVAD-CMK (100 μM) in the presence of 5 ng/ml TGF-beta for 72 h.

### Western Blot Analysis

NRK-52E cells were lysed using the protein lysis buffer radio immunoprecipitation assay containing a 50× protease inhibitor. Equal amounts of total extracted protein were electrophoresed on a 12% SDS-PAGE gel and transblotted onto 0.2 μm polyvinylidene fluoride membranes. After being blocked with 5% non-fat milk powder in tris-buffered saline and 0.1% Tween-20, the membranes were incubated with primary antibodies against NLRP3 (Abcam, 1:1000), high-mobility group box 1 (HMGB1) (CST, 1:1000), alpha-smooth muscle actin (alpha-SMA) (CST, 1:1000), CollagenI (Affinity, 1:1000), IL-1beta (Abcam, 1:1000), GAPDH (Boster, 1:4000), and beta-actin (Boster, 1:1000), followed by the addition of horseradish peroxidase (HRP)-labeled secondary antibodies. Immune-reactive band signals were detected using an enhanced chemiluminescence Western blotting system, and band intensity was measured using ImageJ software.

### Immunohistochemistry Analysis

Rat kidney tissues were paraffin embedded, sectioned, and deparaffinized. After deparaffinization, rehydration, and antigen retrieval, the sections were blocked and incubated with rabbit anti-NLRP3 antibody (NOVUS, 1:100), rabbit anti-HMGB1 antibody (CST, 1:400), mouse anti-MMP9 antibody (Santa, 1:200), mouse anti-MMP2 antibody (Santa, 1:200), or mouse anti-alpha-SMA antibody (CST, 1:200), and were subsequently incubated with goat anti-mouse/anti-rabbit secondary antibody and streptavidin-conjugated HRP. The slices were developed with 3,3’-diaminobenzidine staining and counterstained with hematoxylin. Image Pro Plus 6.0 software was used to measure the percentage of the total positive area.

### Immunofluorescence Microscopy

To detect inflammasome formation and activation in NRK-52E, the immunofluorescence co-localization method was adapted. Goat anti-NLRP3 antibody (1:200, Abcam, Cambridge, MA, United States) and mouse anti-caspase-1 antibody (1:200, Santa Cruz) were used for these experiments. The cells were seeded in culture dishes at a density of 4 × 10^5^ cells per well. After TGF-beta stimulation for 72 h, cells were fixed in 4% paraformaldehyde for 15 min and permeabilized with 0.5% Triton X-100 for 20 min. The cells were then incubated with primary antibody overnight at 4°C, and co-incubated with Alexa Fluor-488- and Alexa Fluor-555-conjugated secondary antibodies (1:200, Invitrogen) for 1.5 h at room temperature. Cells were then washed and visualized with a Zeiss LSM800 microscope (Carl Zeiss, Oberkochen, Germany). Co-localization in cells was analyzed using Image Pro Plus software, and the co-localization coefficient was represented as a Pearson’s correlation coefficient.

### Single-Guide RNA (gRNA) Transfection

NLRP3 was knocked down in NRK-52E by gRNA. gRNA sequences for CRISPR/Cas9 gene editing of coding genes were designed by the CRISPR Design tool^1^, which were synthesized and then inserted into the Bbsl-digested px459 plasmid. The gNlrp3 sequences were 5′-GAAGATTACCCACCCGAGAA-3′. Gene editing in the cells was performed by Lipofectamine 3000 transfection according to the manufacturer’s instructions (Invitrogen, Carlsbad, CA, United States). The transfected cells were incubated in the media with 3 μg/ml puromycin to screen out the gRNA plasmid-containing cells, using Western blotting to analyze the transfection efficiency of cells.

### Statistical Analysis

All data were collected from at least four independent experiments. Results are presented as the mean ± SEM. Two group comparisons were performed using the Student’s *t*-test, and multiple groups were evaluated by one-way ANOVA following the Bonferroni procedure. Differences were defined as statistically significant when *p* < 0.05.

## Results

### Angiotensin II-Induced Renal Fibrosis in Rats

**FIGURE 1:**
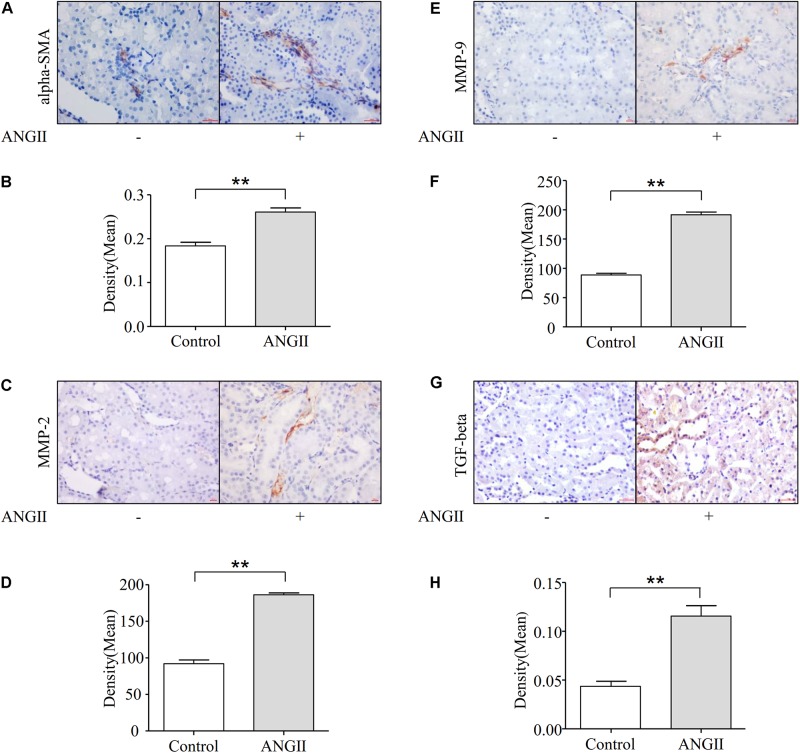
Ang II-induced renal fibrosis in rats. SD rats were treated with Ang II infusion as describe. **(A,B)** Immunohistochemical staining and quantification of alpha-SMA in kidney (*N* = 6). **p < 0.01 vs. Con. **(C,D)** Representative immunohistochemical staining of MMP-2 in kidney (*N* = 6). **p < 0.01 vs. Con. **(E,F)** Immunostaining of MMP-9 in kidney. Representative photomicrographs from Ang II infusion rats (*N* = 6). **p < 0.01 vs. Con. **(G,H)** Immunohistochemical staining and quantification of TGF-beta in kidney (*N* = 6). **p < 0.01 vs. Con.

Angiotensin II is the main effector of RAAS and can exert pro-inflammatory actin, thereby activating fibroblasts and inducing fibrosis of the kidneys. As shown in Figures 1A,B, the subcutaneous infusion of Ang II into nephrectomy rats for 14 days resulted in a considerable increase in the expression of alpha-SMA when compared with that of the control group. The protein expression of MMP-2 and MMP-9 were also increased after treatment with Ang II (Figures 1C–F). Subsequently, we tested the key mediator of tubulointerstitial pathobiology, protein TGF-beta. As shown in Figures 1G,H, positive staining for TGF-beta was significantly increased in rat kidneys undergoing Ang II treatment.

### Angiotensin II Treatment Induces Fibrosis Associated With the Expression of NLRP3 and HMGB1

**FIGURE 2:**
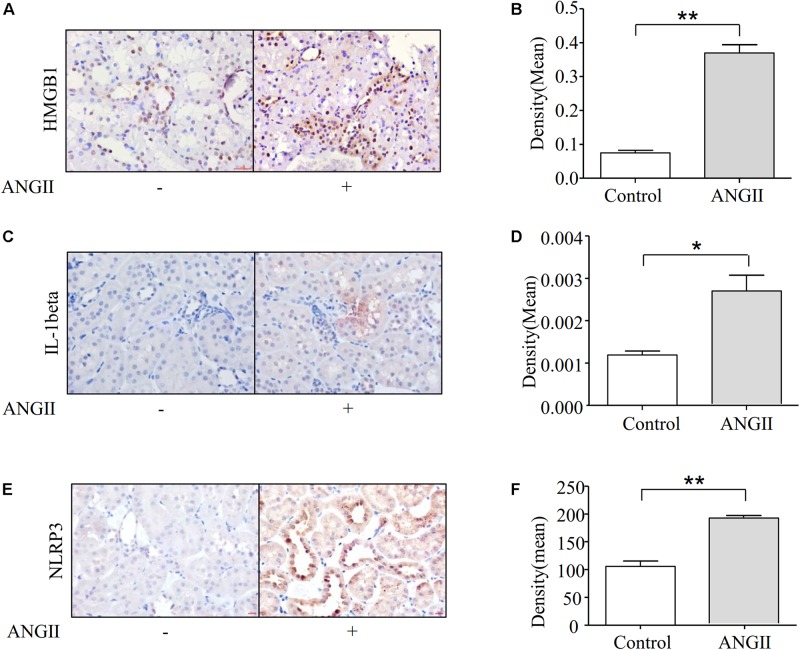
Ang II treatment induces fibrosis associated with the expression of NLRP3 and HMGB1. **(A,B)** Immunohistochemical staining and quantification of HMGB1 in kidney (*N* = 6). **p < 0.01 vs. Con. **(C,D)** Representative immunohistochemical staining of IL-1beta in kidney (*N* = 6). *p < 0.05 vs. Con. **(E,F)** Immunostaining of NLRP3 in the kidneys. Representative photomicrographs from Ang II infusion rats (*N* = 6). **p < 0.01 vs. Con.

A large body of emerging evidence strongly suggested that inflammation plays a pathogenic role in renal fibrosis. Therefore, we examined whether Ang II-induced renal fibrosis is associated with inflammatory cytokine production in kidneys, *in vivo*. Immunohistochemical studies demonstrated that the treatment of rats with Ang II significantly increased the expression of HMGB1 and protein IL-1beta in the kidneys (Figures 2A–D). We further demonstrated that Ang II infusion increased the expression of the NLRP3 protein in rat kidneys as shown in Figures 2E,F.

### Angiotensin II and TGF-Beta Are Capable of Inducing Fibrosis in NRK-52E Cells

**FIGURE 3:**
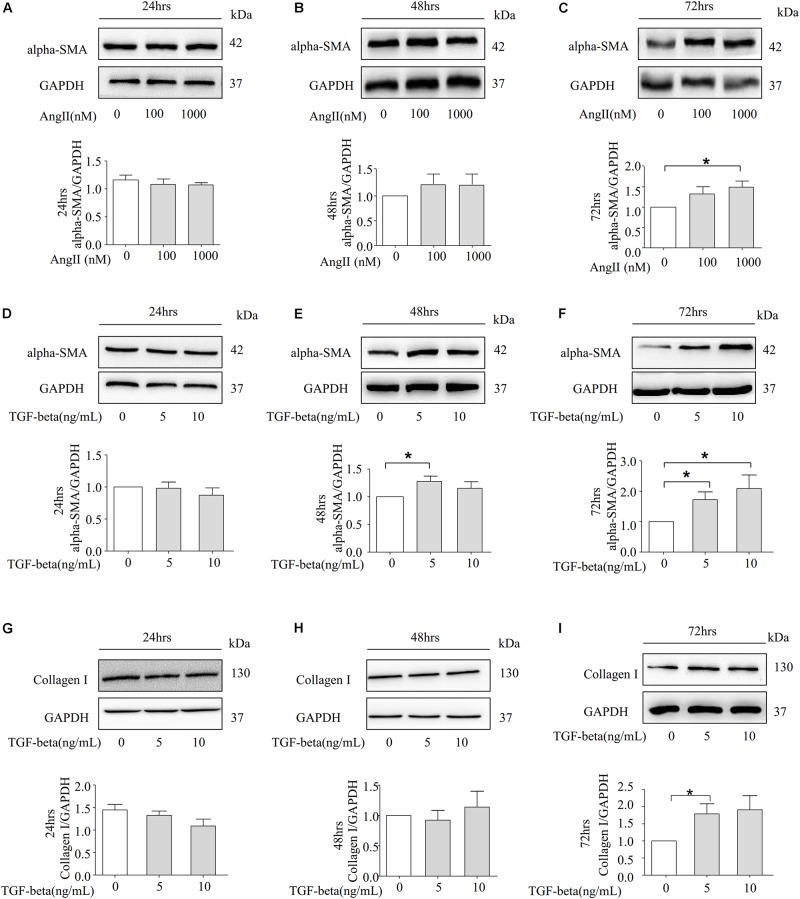
Ang II and TGF-beta are capable of inducing fibrosis in NRK-52E Cells. **(A–C)** Representative Western blot and summarized data showing the effects of Ang II on the expression of alpha-SMA and GAPDH in 24, 48, and 72 h (*N* = 4). *p < 0.05 vs. Ang II 0 nM. **(D–F)** NRK-52E were stimulated with different concentrations of TGF-beta (0, 5, 10 ng/ml) for 24, 48, and 72 h and total proteins that analyzed by Western blot using antibodies against alpha-SMA (*N* = 4). *p < 0.05 vs. TGF-beta 0 ng/ml. **(G–I)** Representative Western blot and summarized data of CollagenI and GAPDH in 24, 48, and 72 h (*N* = 4). *p < 0.05, vs. TGF-beta 0 ng/ml.

Our data show that Ang II can promote the protein expression of alpha-SMA in rat kidneys. Subsequently, we detected the level of alpha-SMA across different time points with Ang II stimulation in NRK-52E cells. However, a significant difference was observed in the alpha-SMA expression between the NRK-52E cells treated with Ang II and those without treatment in 72 h (Figures 3A–C). We also detected the protein level of alpha-SMA in the presence of TGF-beta. Notably, the addition of TGF-beta induced a dose-dependent increase in alpha-SMA protein expression in 72 h (Figures 3D–F). We further detected the expression of another fibrotic marker, CollagenI, and as the data shows in Figures 3G–I, the Western blot study indicates that the protein level of CollagenI was significantly increased after stimulation by TGF-beta in 72 h.

### TGF-Beta Induced the Expression of NLRP3 in NRK-52E Cells

**FIGURE 4:**
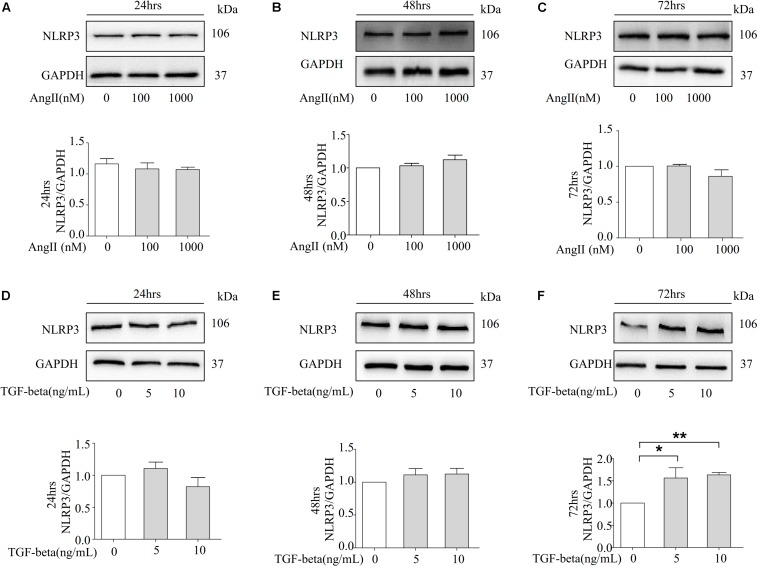
TGF-beta induced the expression of NLRP3 in NRK-52E cells. **(A–C)** Representative Western blot and summarized data showing the effects of Ang II on the expression of NLRP3 and GAPDH in 24, 48, and 72 h (*N* = 4). *p < 0.05 vs. Ang II 0 nM. **(D–F)** NRK-52E were stimulated with different concentrations of TGF-beta (0, 5, 10 ng/ml) for 24, 48, and 72 h and total proteins that analyzed by Western blot using antibodies against NLRP3 (*N* = 4). *p < 0.05, **p < 0.01 vs. TGF-beta 0 ng/ml.

NRK-52E cells are thought to belong to the proximal tubular epithelial cell line in normal rat kidneys due to the patterns of collagen production, the secretion of C-type natriuretic peptides, and the expression of epidermal growth factor receptors. We further examined whether Ang II induces the expression of NLRP3 proteins in NRK-52E cells. However, Ang II failed to increase the protein level of NLRP3 at 100 and 1000 nM over 24, 48, and 72 h (Figures 4A–C). Our animal studies found that Ang II played a role in the protein level of TGF-beta. Therefore, we investigated whether TGF-beta stimulates NRK-52E cells to increase the expression of NLRP3. As shown in Figures 4D–F, the Western blot analysis revealed that the NLRP3 protein was significantly upregulated after 72 h of stimulation.

### NLRP3 Inflammasomes Promote TGF-Beta-Induced Epithelial–Mesenchymal Transition via HMGB1

**FIGURE 5:**
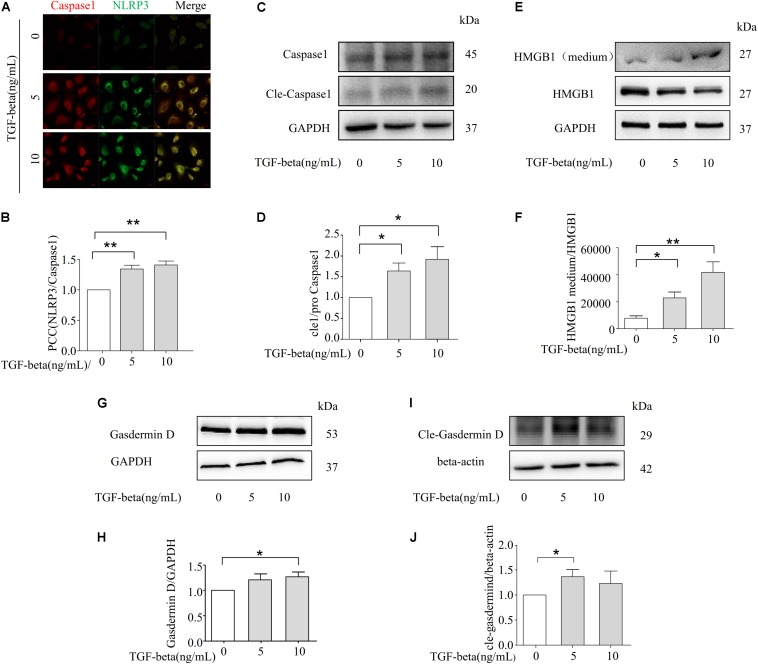
NLRP3 inflammasome promotes TGF-beta-induced epithelial–mesenchymal transition (EMT) via HMGB1. **(A,B)** NLRP3/CASPASE-1 was identified by confocal microscopy to determine the formation of inflammasome (*N* = 4). **(C,D)** Representative Western blot and summarized data showing the expression of Caspase-1, cleave-caspase-1, and GAPDH (*N* = 4). *p < 0.05 vs. TGF-beta 0 ng/ml. **(E,F)** The expression of HMGB1 in studied by Western blotting. *p < 0.05, **p < 0.01 vs. TGF-beta 0 ng/ml. **(G,H)** and **(I,J)** Representative Western blot and summarized data showing the expression of Gasdermin D and cle-Gasdermin D (*N* = 4). *p < 0.05 vs. TGF-beta 0 ng/ml.

We next examined whether TGF-beta-induced EMT is associated with the formation and activation of NLRP3 inflammasomes. As the data shows in Figures 5A,B, the co-localization of NLRP3/CASPASE-1 was increased with the stimulation of TGF-beta. Additionally, the expression of cleaved-caspase-1 was upregulated with the treatment of TGF-beta, indicating that the NLRP3 inflammasome had been activated (Figures 5C,D). Given that the HMGB1 protein is located in the nucleus, activation of the inflammasome can induce HMGB1 translocation from the nucleus to the cytoplasm, along with a subsequent release to the extracellular matrix to promote the progression of pyroptosis (Qin et al., 2006). Therefore, we detected the released HMGB1 in the culture medium of NRK-52E. HMGB1 in the supernatant was found to increase with TGF-beta stimulation, while the HMGB1 expression of intracellular proteins was significantly decreased (Figures 5E,F). More importantly, Gasdermin D is related to the release of HMGB1 (Davis et al., 2019), and the expression of caspase-1 genetic substrate Gasdermin D and its cleavage were also increased in the presence of TGF-beta (Figures 5G–J). These findings demonstrate the involvement of HMGB1 in TGF-beta-induced fibrosis in NRK-52E cells.

### Inhibition of the NLRP3 Inflammasome Alleviates TGF-Beta-Induced Fibrosis in NRK-52E Cells

**FIGURE 6:**
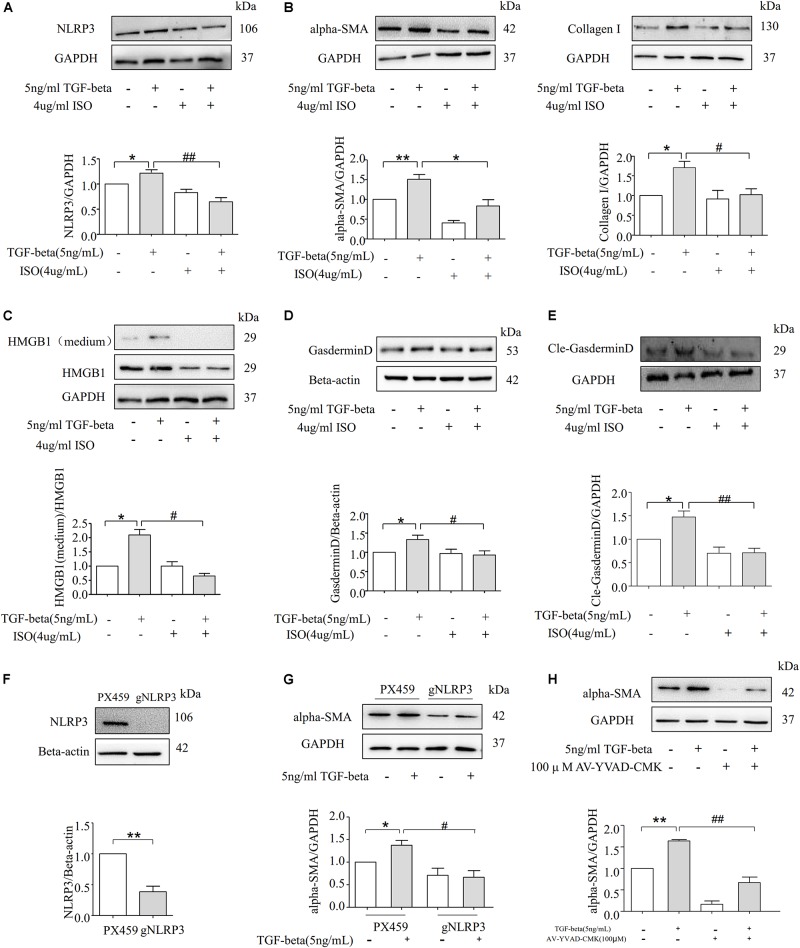
Inhibition of NLRP3 inflammasome alleviates TGF-beta-induced fibrosis in NRK-52E Cells. **(A)** Western blot analysis for NLRP3 protein level with or without NLRP3 inhibitor (*N* = 4). *p < 0.05 vs. TGF-beta 0 ng/ml. **(B)** Representative Western blot and summarized data showing the expression of alpha-SMA, CollagenI with or without NLRP3 inhibitor (*N* = 4). *p < 0.05 vs. TGF-beta 0 ng/ml. **(C)** The expression of HMGB1 in studied by Western blotting. *p < 0.05 vs. TGF-beta 0 ng/ml. **(D)** The documents and summarized data showing the effects on the NLRP3 inflammasome inhibition on the expression of GasderminD and β-actin (*N* = 4). *p < 0.05 vs. TGF-beta 0 ng/ml. **(E)** The documents and summarized data showing the effects on the NLRP3 inflammasome inhibition on the expression of cle-Gasdermin D and GAPDH. *p < 0.05 vs. TGF-beta 0 ng/ml. **(F)** NLRP3 gene knockdown succeeded. **(G)** Representative Western blot and summarized data show the expression of alpha-SMA with scramble or NLRP3 gRNA plasmids. *p < 0.05 vs. TGF-beta 0 ng/ml. **(H)** Western blot analysis for alpha-SMA protein level with or without Caspase-1 inhibitor AV-YVAD-CMK (*N* = 4). *p < 0.05 vs. TGF-beta 0 ng/ml. **p < 0.01 vs. TGF-beta 0 ng/ml. ^#^*p* < 0.05 vs. TGF-beta 5 ng/ml. ^##^*p* < 0.01 vs. TGF-beta 5 ng/ml.

To further confirm the role of NLRP3 in TGF-beta-induced EMT, NRK-52E cells in the presence of vehicle or NLRP3 inhibitor (ISO, 4.4 μM) were then stimulated with or without TGF-beta for 72 h. As shown in Figure 6A, the inhibition of NLRP3 by ISO markedly attenuated the expression of the NLRP3 protein in TGF-beta-induced NRK-52E cells. Moreover, TGF-beta-induced upregulation of alpha-SMA and CollagenI were almost entirely prevented in NRK-52E cells treated with ISO, as shown in Figure 6B. Consistent with these findings, TGF-beta induced the release of HMGB1 and was inhibited by the NLRP3 inhibitor (Figure 6C), and the expression of Gasdermin D and its cleavage form were blocked in the presence of the NLRP3 inhibitor (Figures 6D,E). These results suggest that NLRP3-dependent HMGB1 release plays an important role in TGF-beta-induced fibrosis in NRK-52E cells. The gene of NLRP3 was knocked down in NRK-52E by gRNA, and the silencing efficiency was shown by Western blot analysis in Figure 6F. Compared with the scramble group, the TGF-beta stimulation of the knockdown group showed that there was no significant change in the expression of alpha-SMA (Figure 6G). And the Caspase-1 inhibitor AV-YVAD-CMK (100 μM) also significantly blocked a TGF beta-induced increase in the expression of alpha-SMA (Figure 6H).

## Discussion

The present study demonstrated that the NLRP3 inflammasomes mediate TGF-beta induced renal fibrosis. Our results verified that NLRP3 inflammasome aggregation and activation promote the release of HMGB1 through Gasdermin D, and it also mediates the EMT induced by TGF-beta. Suppression of the NLRP3 inflammasome via isoliquiritigenin reduces the release of HMGB1 and may ameliorate fibrosis.

Angiotensin II is a vasoactive peptide of the RAAS, which can activate interstitial fibroblasts and tubular cells, ultimately resulting in the development of fibrosis in patients with CKD (Balakumar et al., 2019). It has been largely demonstrated that TGF-beta serves as a key downstream mediator in Ang II-induced chronic renal fibrosis (Iekushi et al., 2011). Fibrosis resulting in the excessive synthesis and accumulation of interstitial matrix proteins could be induced by Ang II via the upregulation of TGF-beta (Ehanire et al., 2015a; Ding et al., 2019). Consistent with these findings, our data showed that following Ang II infusion, the expression of fibrotic markers alpha-SMA, MMP-2, and MMP-9 were all significantly increased (Figures 1A–F). We next examined TGF-beta levels in rat kidneys, and immunohistochemistry studies demonstrated that the treatment of rats with Ang II markedly increased the expression of TGF-beta (Figures 1G,H). The NLRP3 protein is the main functional component of inflammasomes, which has been discovered to mediate a wide variety of diseases, such as diabetes, gouty arthritis, microbial infection, atherosclerosis, and non-alcoholic steatohepatitis (Groslambert and Py, 2018). Moreover, it has been reported that the NLRP3 inflammasome plays a crucial role in the process of fibrosis in CKD progression (Vilaysane et al., 2010; Chang et al., 2014; Lorenz et al., 2014).

Our data show that Ang II infusion induced the activation of NLRP3 inflammasomes in rat kidneys (Figures 2A–D). Previous studies have revealed the role of NLRP3 inflammasomes in mediating the release of HMGB1 (Lamkanfi et al., 2010; Chen et al., 2015; Zhou et al., 2019). Several reports have identified that HMGB1, a member of the high mobility group nuclear protein family, acts as a nuclear homeostasis DNA-binding protein (Paudel et al., 2019). Extended stimulation triggers HMGB1 to translocate from the nucleus into the cytosol and eventually releasex into the extracellular matrix, promoting the secretion of proinflammatory cytokines and mediating the process of fibrosis progression. Previous studies demonstrated that recombinant human HMGB1 induces the EMT in human proximal tubular epithelial cells, including alterations in epithelial morphology, increasing the expression of alpha-SMA, and reducing the protein level of E-cadherin (Boor et al., 2010). Furthermore, the expression of HMGB1 was analyzed by evaluating the positive areas of immunohistochemistry. Our data shows that with the increased level of NLRP3, the expression of HMGB1 was also upregulated (Figures 2E,F), indicating that NLRP3 inflammasomes mediated the release of HMGB1, which is associated with the progression of fibrosis. Several reports have identified that inhibition of the TGF-beta receptor can prevent the phenotype changes of EMT and fibrogenesis induced by Ang II (Burns et al., 2010). However, the underlying mechanisms of TGF-beta in promoting the progression of fibrosis have not been elucidated. Therefore, determining whether NLRP3 inflammasomes play a crucial role in TGF-beta-mediated renal fibrosis is required before effective therapeutic targets can be identified.

Recent studies have confirmed that TGF-beta stimulation was found to induce NLRP3 abundance in a Smad3-dependent manner, and NLRP3 can also promote the progression of renal tubular EMT via the enhancement of TGF-beta signaling and the activation of R-Smad (Lorenz et al., 2014; El-Deeb et al., 2019). In the present study, compared to Ang II stimulation (Figures 3A–C), the NRK-52E cells are more sensitive to TGF-beta, in which the fibrotic marker alpha-SMA and CollagenI were significantly upregulated in 72 h (Figures 3D–I). Importantly, our results indicate that the role of Ang II on increasing the expression of NLRP3 is not obvious in NRK-52E cells (Figures 4A–C). The activation of transcription factors NF-κB is associated with the regulation of NLRP3 transcription, and we measured the protein level of NLRP3 transcriptional factor NF-κB and p-NF-κB by Western blot. As data shown in Supplementary Figure 2B, there was no statistics significance compared to the control group in the protein level of NF-κB and p-NF-κB. However, the expression of cle-caspase-1 was upregulated in the presence of 1000 nM Ang II in 72 h (Supplementary Figure 2A), suggesting that Ang II could not trigger the NLRP3 transcription but can activate the NLRP3 inflammasome after 72 h of stimulation in NRK-52E cells. But in the presence of TGF-beta, the protein level of NLRP3 was significantly upregulated after 72 h (Figures 4D–F).

The present study further examined the relationship between NLRP3 inflammasomes and TGF-beta-induced EMT in NRK-52E cells. Previous work demonstrated that the marker of inflammasome activation is the conversion of caspase-1, and in turn the cleavage of caspase-1 acts on the substrates pro-IL-1beta and pro-IL-18 to promote the maturation of cytokines IL-1beta and IL-18 (Groslambert and Py, 2018). In our study, our data showed that TGF-beta can promote the activation and formation of NLRP3 inflammasome (Figures 5A,B). We found that in the presence of TGF-beta, pro-caspase-1 is converted to its active form, cleave-caspase-1, in NRK-52E cells as demonstrated through our Western blot analysis (Figures 5C,D). These results suggest that TGF-beta can induce the activation of NLRP3 inflammasomes. ROS under physiological conditions serves as mediators in controlling cell growth and proliferation, which is integral components of multiple cellular signaling. Excessive ROS causes oxidative stress, leading to conformational changes caused by oxidation of proteins, which plays an important role in the regulation of NLRP3 inflammasome activation (Han et al., 2018; Yang et al., 2019). In order to further investigate the upstream mechanism, we studied the relationship between TGF beta-NLRP3 inflammasome signaling and ROS production in NRK-52E cells. According to our results (Supplementary Figure 1), TGF-beta stimulation can promote the release of ROS in different doses, which suggests that the overproduced ROS by TGF-beta may further cause the activation of NLRP3 inflammasome. Chen et al. (2013) reported that high glucose levels induced NLRP3 inflammasome activation through ATP-P2X4 signaling, regulating the maturation and secretion of IL-1β and IL-18 cytokines in diabetic nephropathy. Furthermore, IL-18 serves as a key mediator in obstruction-induced tubulointerstitial fibrosis and EMT (Bani-Hani et al., 2009), which confirms that the production of NLRP3 inflammasomes plays an important role in mediating renal dysfunction and fibrosis. Several reports indicate that NLRP3 inflammasome expression is primarily attributed to the increased release of HMGB1 (Lu et al., 2013), as the genetic deletion of inflammasome components severely reduces the release of HMGB1 during endotoxemia or bacteremia (Lu et al., 2013). Our data demonstrate that the protein expression of HMGB1 in extracellular space is significantly increased with TGF-beta stimulation (Figures 5E,F). HMGB1 is well documented to be involved in the progression of fibrosis. With the acute exacerbation of idiopathic pulmonary fibrosis, the level of HMGB1 is severely increased in the lungs and circulatory system of patients (Yamaguchi et al., 2019). Exogenous HMGB1 treatment can promote the proliferation of human dermal fibroblasts and act as a profibrotic molecule to upregulate collagen synthesis and deposition (Lee et al., 2018). However, HMGB1 lacks a classic secretion signal like the endoplasmic reticulum–Golgi exocytosis pathway, thus the mechanism by which HMGB1 is released from the cytoplasm needs to be identified. Gasdermin D is a newly identified genetic substrate for inflammatory caspases, which is known to serve as a key downstream mediator for pyroptosis of the inflammasome signaling pathways (Evavold et al., 2018). Once cleaved by an activated caspase, Gasdermin D allows its N-terminal domain to associate with membrane lipids and form membrane pores, leading to cytokine secretion and programed cell death (Zhao et al., 2018). This cleavage event enables the Gasdermin D pore-forming amino-terminal domain to oligomerize and permeabilize the cell plasma membrane, which causes a rapid loss of plasma membrane integrity and ultimately results in cellular lysis (Kanneganti et al., 2018). After permeabilization, pyroptotic cells are thought to rupture, releasing soluble inflammatory intracellular content with characteristic cell swelling. This process is thought to promote the extracellular release of nuclear HMGB1 during infections (Lamkanfi et al., 2010). While the secretion of lactate dehydrogenase (LDH), HMGB1, and IL-1β occurs without rupture, HMGB1 can be released to the extracellular matrix via passive diffusion through Gasdermin D pores (Davis et al., 2019; Miao et al., 2019), and living cells are capable of releasing inflammasome related cytokines via the Gasdermin D pore without the development of pyroptosis (Xia et al., 2019). Another novel finding is that GPX4 deficiency increases lipid peroxidation, thus exacerbating GSDMD-mediated pyroptosis in macrophages as well as septic lethality in mice. Depletion of GPX4 increased GSDMD-N formation, and consequently induced the release of matured IL-1β and HMGB1 in LPS-primed BMDMs (Kang et al., 2018). We further examined the relationship between Gasdermin D and TGF-beta induced HMGB1 release. Our results show that TGF-beta can upregulate the expression of Gasdermin D and its cleavage form (Figures 5G–J), and we assumed that the release of HMGB1 is activated via TGF beta-1-induced Gasdermin D cleavage.

The present study further determined whether the NLRP3 inflammasome inhibitor ISO can ameliorate TGF-beta induced EMT. Our results demonstrate that the EMT markers alpha-SMA and CollagenI were significantly attenuated in ISO-treated NRK-52E cells (Figure 6B), suggesting that NLRP3 inflammasomes play a crucial role in TGF-beta induced EMT. In addition, the level of HMGB1 in the cytoplasm and the extracellular matrix both decreased (Figure 6C), indicating that the blockage of the NLRP3 protein can abrogate the phenomenon of HMGB1-mediated EMT. Furthermore, our data shows that after blocking the NLRP3 inflammasome, TGF-beta failed to upregulate the protein level of Gasdermin D and cle-Gasdermin D (Figures 6D,E). Deletion of NLRP3 by gRNA and using Caspase-1 inhibitor AV-YVAD-CMK could attenuate the expression of the alpha-SMA protein in TGF-beta-induced NRK-52E cells (Figures 6F–H).

**FIGURE 7:**
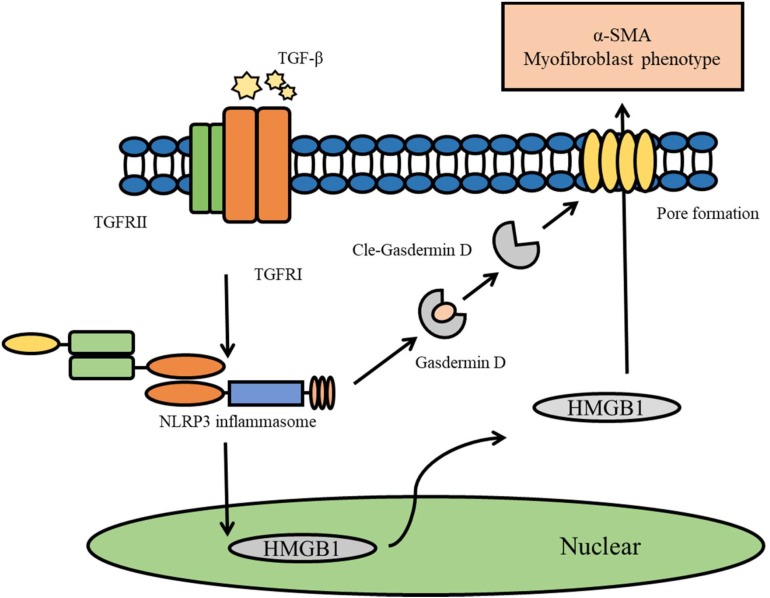
Diagram representing the putative mechanism by which TGF-beta induce the activation of NLRP3 inflammasome to release HMGB1 through GasderminD to promote EMT and fibrosis.

In summary, this study revealed that TGF-beta induces the activation of the NLRP3 inflammasome, and that the NLRP3 inflammasome-mediated HMGB1 release through Gasdermin D plays an important role in the process of EMT in NRK-52E cells (Figure 7). Our results provide novel findings that indicate that HMGB1 is a key mediator associated with NLRP3 inflammasome-mediated fibrogenesis.

## Data Availability Statement

All datasets generated for this study are included in the article/Supplementary Material.

## Ethics Statement

The animal study was reviewed and approved by the Animal Ethics Committee of Guangzhou University of Chinese Medicine.

## Author Contributions

KZ and CF performed the research and drafted the manuscript. DC, YZ, RZ, and LZ analyzed the data. YCa and JZ critically revised the manuscript for important intellectual content. CL, YCh, and HL designed the study and approved the final version of the manuscript which to be submitted.

## Conflict of Interest

The authors declare that the research was conducted in the absence of any commercial or financial relationships that could be construed as a potential conflict of interest.

## Footnotes

1 http://crispr.mit.edu/

## Abbreviations

Ang II: angiotensin II
CKD: chronic kidney disease
EMT: epithelial–mesenchymal transition
FBS: fetal bovine serum
UUO: unilateral ureteral obstruction.
