# Supplementary material for: Contribution of TGF-Beta-Mediated NLRP3-HMGB1 Activation to Tubulointerstitial Fibrosis in Rat With Angiotensin II-Induced Chronic Kidney Disease
Source: Front Cell Dev Biol. 2020 Feb 5;8:1. doi: 10.3389/fcell.2020.00001 (PMC7012792; doi:10.3389/fcell.2020.00001)
Supplement: Supplementary file 1 [file Data_Sheet_1.pdf]

Supplementary Figure 1

A

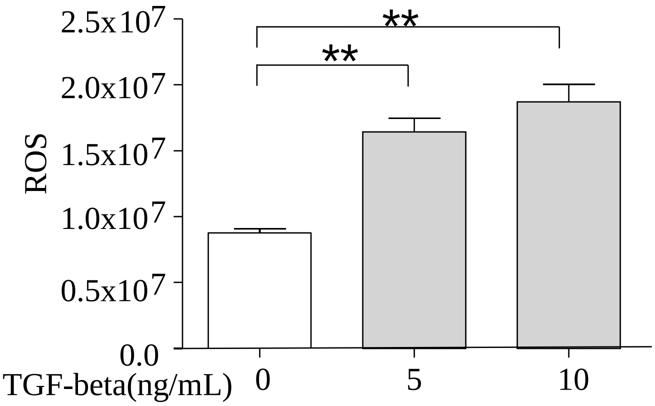

Supplementary Figure 1 TGF-beta increased the level of ROS in NRK-52E cells**.** summarized data showing the effects of TGF-beta on the level of ROS in 72h (N=6). *$p < 0.05$, **$p < 0.01$ vs. TGF-beta 0ng/ml.

Supplementary Figure 2

A

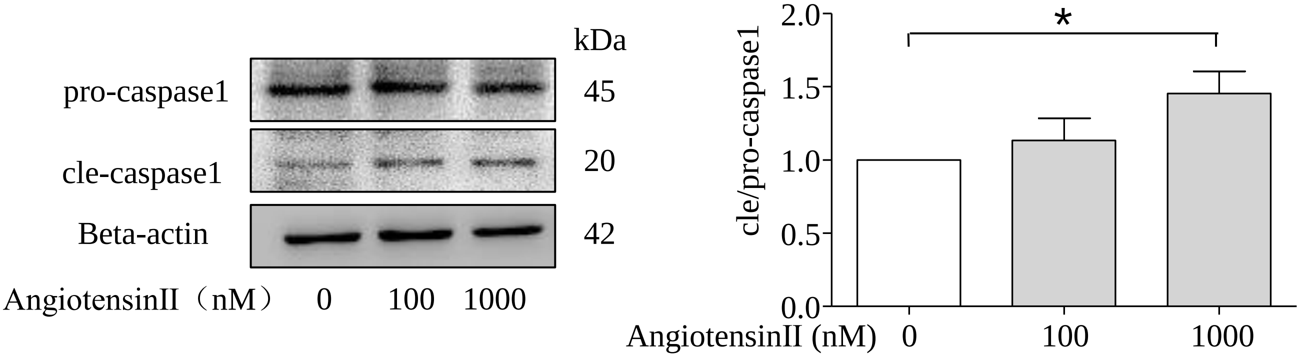

B

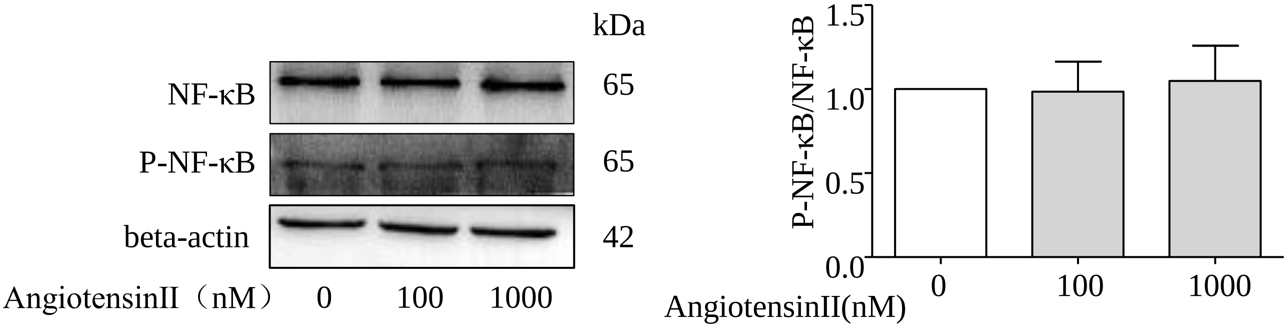

Supplementary Figure 2 the effects of AngiotensinII on NLRP3 inflammasome activation and NLRP3 transcription.(A)Representative Westernblot and summarized data showing the expression of Caspase1,cleave-caspase1 and beta-actin (N=4).*p $<$ 0.05 vs. AngII 0nM.(B)Representative Westernblot and summarized data showing the expression of NF-κB, p-NF-κB and beta-actin (N=4).
